# Nasal swab samples and real-time polymerase chain reaction assays in community-based, longitudinal studies of respiratory viruses: the importance of sample integrity and quality control

**DOI:** 10.1186/1471-2334-14-15

**Published:** 2014-01-09

**Authors:** Asma N Alsaleh, David M Whiley, Seweryn Bialasiewicz, Stephen B Lambert, Robert S Ware, Michael D Nissen, Theo P Sloots, Keith Grimwood

**Affiliations:** 1 Queensland Children’s Medical Research Institute, The University of Queensland, Brisbane Queensland 4029, Australia; 2 Queensland Paediatric Infectious Disease Laboratory, Royal Children’s Hospital, Brisbane Queensland 4029, Australia; 3 Department of Microbiology, College of Science, King Saud University, Riyadh 11451, Saudi Arabia; 4 Queensland Health Communicable Diseases Branch, Queensland Health, Brisbane Queensland 4006, Australia; 5 School of Population Health, The University of Queensland, Brisbane Queensland 4006, Australia; 6 Microbiology Division, Pathology Queensland Central Laboratory, Brisbane Queensland 4006, Australia

**Keywords:** Nasal swab, Respiratory virus, Real-time polymerase chain reaction, Quality control, Mould, Community-based study

## Abstract

**Background:**

Carefully conducted, community-based, longitudinal studies are required to gain further understanding of the nature and timing of respiratory viruses causing infections in the population. However, such studies pose unique challenges for field specimen collection, including as we have observed the appearance of mould in some nasal swab specimens. We therefore investigated the impact of sample collection quality and the presence of visible mould in samples upon respiratory virus detection by real-time polymerase chain reaction (PCR) assays.

**Methods:**

Anterior nasal swab samples were collected from infants participating in an ongoing community-based, longitudinal, dynamic birth cohort study. The samples were first collected from each infant shortly after birth and weekly thereafter. They were then mailed to the laboratory where they were catalogued, stored at -80°C and later screened by PCR for 17 respiratory viruses. The quality of specimen collection was assessed by screening for human deoxyribonucleic acid (DNA) using endogenous retrovirus 3 (ERV3). The impact of ERV3 load upon respiratory virus detection and the impact of visible mould observed in a subset of swabs reaching the laboratory upon both ERV3 loads and respiratory virus detection was determined.

**Results:**

In total, 4933 nasal swabs were received in the laboratory. ERV3 load in nasal swabs was associated with respiratory virus detection. Reduced respiratory virus detection (odds ratio 0.35; 95% confidence interval 0.27-0.44) was observed in samples where the ERV3 could not be identified. Mould was associated with increased time of samples reaching the laboratory and reduced ERV3 loads and respiratory virus detection.

**Conclusion:**

Suboptimal sample collection and high levels of visible mould can impact negatively upon sample quality. Quality control measures, including monitoring human DNA loads using ERV3 as a marker for epithelial cell components in samples should be undertaken to optimize the validity of real-time PCR results for respiratory virus investigations in community-based studies.

## Background

Acute respiratory infections (ARIs) caused by viruses are the most common illnesses experienced by all age groups. ARIs are particularly important during early life as infants have the highest infection rates and they can transmit infectious agents to other household members [1]. Recently introduced molecular-based diagnostic techniques have much improved sensitivity compared with previous classical culture and phenotypic-based methods and have led to the discovery of new respiratory viruses [2]. However, contemporary studies employing these new techniques have often used convenience samples obtained from patients admitted to hospital or attending Emergency Department clinics [3-5]. Understanding more fully the ARI disease burden in the community is important for developing public health interventions, such as vaccination programs [6], and for understanding the role respiratory viruses may play in the pathogenesis of certain chronic pulmonary disorders, such as asthma [7-9]. This has led to the instigation of community-based studies. Such studies do however have some logistical challenges, particularly concerning respiratory sample collection and transport. Most studies have relied upon clinic or home visits by trained healthcare workers to collect specimens during an ARI episode, which imposes restrictions upon busy families and may lead to biased disease estimates and specimen availability [10-12]. Cost and feasibility of using healthcare workers are also important when large longitudinal, community-based cohort studies, involving frequent specimen collections, are planned. To help address these limitations, we and others have begun testing parent-collected, anterior nasal swab specimens that have been transported to the research laboratory using the standard mail [13-16]. This approach is considered to be safe, convenient and cost-effective [17].

Importantly, when using highly sensitive polymerase chain reaction (PCR) assays the detection rates for respiratory viruses are similar in both anterior nasal swab specimens and samples collected by the more traditional method of nasopharyngeal aspiration [18,19]. Building on this information, later studies have also shown that PCR testing for respiratory viruses provided similar results for parent-collected anterior nasal swab specimens and either nasal swab or nasoparyngeal aspirates collected by healthcare professionals [16,17]. Other studies examining sample transport have also shown that mailing swabs at ambient temperature has limited or no impact on respiratory virus detection by PCR [14,20,21], although investigating further the effects of transporting samples for extended periods and at higher temperatures was highlighted in one study [20].

The observational research in childhood infectious diseases (ORChID) project is a longitudinal, community-based, dynamic birth cohort study, which seeks to describe the nature and timing of respiratory viruses detected in Australian children during the first 2-years of life [22]. The study commenced in late 2010 and involves parents collecting and mailing nasal swabs weekly to the research laboratory for PCR-based respiratory virus screening. During the first year mould was seen in some samples as they arrived in the laboratory and we became concerned about the impact of this contaminant upon sample integrity. Therefore, as part of the ORChID study, we undertook a broader investigation of sample quality, examining collection and transportation, and how these impact on respiratory virus detection. Our objectives were first to determine the quality of specimen collection by testing for the presence of human DNA (endogenous retrovirus3; ERV3) and then to investigate the effects of sample quality and the presence of visible mould in samples reaching the laboratory upon PCR performance.

## Methods

### The cohort

Briefly, as part of ORChID, families expecting a healthy term baby were recruited antenatally at either the publically funded Royal Brisbane and Women’s Hospital or the North West Private Hospital, in Brisbane, Australia, a subtropical city of more than 2 million inhabitants [22].

### Ethics statement

The Human Research Ethics Committees of the Children’s Health Queensland Hospital and Health Service, the Royal Brisbane and Women’s Hospital and the University of Queensland approved the study. Parents/caregivers of each baby provided written, informed consent at the time of enrolment into the study.

### Sample collection

Parents were asked to record from birth a daily symptom diary and to collect anterior nasal swab samples every week until their infant’s second birthday. Instructions on sample collection were provided at the initial visit by research staff who also demonstrated the technique by undertaking the initial nasal swab specimen shortly after delivery of the newborn baby. In addition, parents were given written instructions on how to collect nasal swab specimens. They also received regular text messages, emails or telephone calls as means of research staff keeping in contact with participating families. Regular supplies of sterile rayon swabs (Virocult, MW950, Medical Wire & Equipment, England) were provided, which were rotated against the internal anterior walls of both nostrils and then placed in the provided transport tube that contained a viral transport media-soaked foam pad in the base. Parents were instructed to squeeze the foam pad to release the fluid and bathe the top of the swab. Ideally within 24-hours of collection, the nasal swabs were then sent by regular postal mail (in accordance with Australia Post regulations [23]) at ambient temperature to our research laboratory where they were stored at -80°C until analysis.

### DNA extraction and quality control measures

Nasal swabs were vortexed in 2 mL of phosphate buffered saline from which 200 μL was spiked with 5 μL of equine herpes virus-1 (EHV1) culture supernatant, which served as an extraction and inhibition control agent, before nucleic acid was extracted using the CAS1820 XtractorGene automated system (Qiagen-Australia) according to the manufacturer’s instructions. The final volumes of specimen extracts were 150 μL/specimen eluted in 96 well racks (Matrix, Thermo Scientific, Australia). For each run (96 extracts/run), extracts were tested using a duplex real-time PCR assay for EHV1 and ERV3 in the following reaction compositions; 10pmoles of each primer, 4pmoles of each probe (Table 1), 10 μL of SensiMix II Probe PCR Mix (Bioline, Australia) and 2 μL of extract in a 20 μL final reaction. Cycling conditions used for amplification were: initial hold at 10 min at 95°C; followed by 45 cycles of 30 sec at 95°C and 60 sec at 60°C. The EHV1 component was performed as an extraction and inhibitor control as described previously [24]**,** while ERV3 was used as a marker to evaluate the quality of nasal swab sample collection [25]. Briefly, the samples were considered to have failed the EHV1 component (ie. failed extraction or possessed PCR inhibitors) if the EHV1 real-time PCR cycle threshold (Ct) results for individual samples were more than two standard deviations from the mean value of all samples, which for this study was calculated to be approximately 30 cycles [24].

**Table 1:** Oligonucleotide primers for equine herpes virus-1 (EHV 1) and endogenous retrovirus 3 (ERV3) used for samples quality control

**Name**	**Sequence**	**Reference**
EHV1-F	GATGACACTAGCGACTTCGA	[24]
EHV1-R	CAGGGCAGAAACCATAGACA	
EHV1-TM	Quasar-670-TTTCGCGTGCCTCCTCCAG-bhq2	
ERV3-F	CATGGGAAGCAAGGGAACTAATG	[25]
ERV3-R	CCCAGCGAGCAATACAGAATTT	
ERV3-TM	Fam-TCTTCCCTCGAACCTGCACCATCAAGTCA-bhq1	

Sequences are listed 5` to 3`.

### Respiratory virus screening

Samples that passed EHV1 DNA extraction quality control testing were screened for respiratory viruses using previously optimized and described PCR and reverse transcriptase PCR assays. Virus testing assays included: rhinovirus (RV) [26], influenza viruses (A and B) [27], respiratory syncytial viruses (A and B) [28], parainfluenza viruses (1–3) [29], human adenoviruses [22], human metapneumovirus [30], human coronaviruses (OC43, HKU1, 229E, and NL63) [31,32], human bocavirus [33] and human polyomaviruses (WUPyV and KIPyV) [34]. For all viruses, except RV, samples were tested in a 10 × 10 pooled format. Briefly, aliquots of the sample extracts were pooled using the CAS-1200 liquid handling system (Qiagen-Australia) and pools tested for the presence of respiratory viruses. For positive pools, individual sample extracts were then tested to confirm positivity. RV screening was performed on individual sample extracts, and not on the pooled extracts, as the number of expected positive samples was considered too high for there to be any benefits from pooling.

### Fungal testing

During the initial phases of the study, mould was observed growing on a small number of nasal swabs at the time of their arrival at the Laboratory. In light of this observation, before extraction all swabs were inspected visually for mould and were assigned a semi-qualitative score according to a sliding scale (0 to 3), whereby 0 = no mould observed, 1 = low, 2 = medium, and 3 = high levels of visible mould present. DNA sequencing was used to identify the type of fungi present on a subset of swabs exhibiting varying degrees of visible mould growth (10 swabs where no mould was seen, and 20 each where low, medium and high levels, respectively, of mould contamination was present).

PCR amplification of a fungal internal transcribed spacer (ITS) region was performed using 10 pmoles of forward and reverse primers (ITS1 forward primer TCCGTAGGT GAACCTGCGG and ITS4-reverse primer TCCTCCGCTTA TTGATATGC [35], 25 μL of Qiagen SYBR master mix (Qiagen, Australia) and 5 μL of template in a total 50 μL reaction mix. Cycling was performed using the following conditions: 95°C for 15 min, 45 cycles of 95°C for 30 sec, 50°C for 30 sec and 72°C for 60 sec and a melting step of 60-95°C at the end of the thermal cycling. PCR products were examined by gel electrophoresis using a 2% agarose gel and sent to the Australian Genome Research Facility (The University of Queensland, Brisbane) for automated sequencing.

### Exclusion criteria

**Figure 1:**
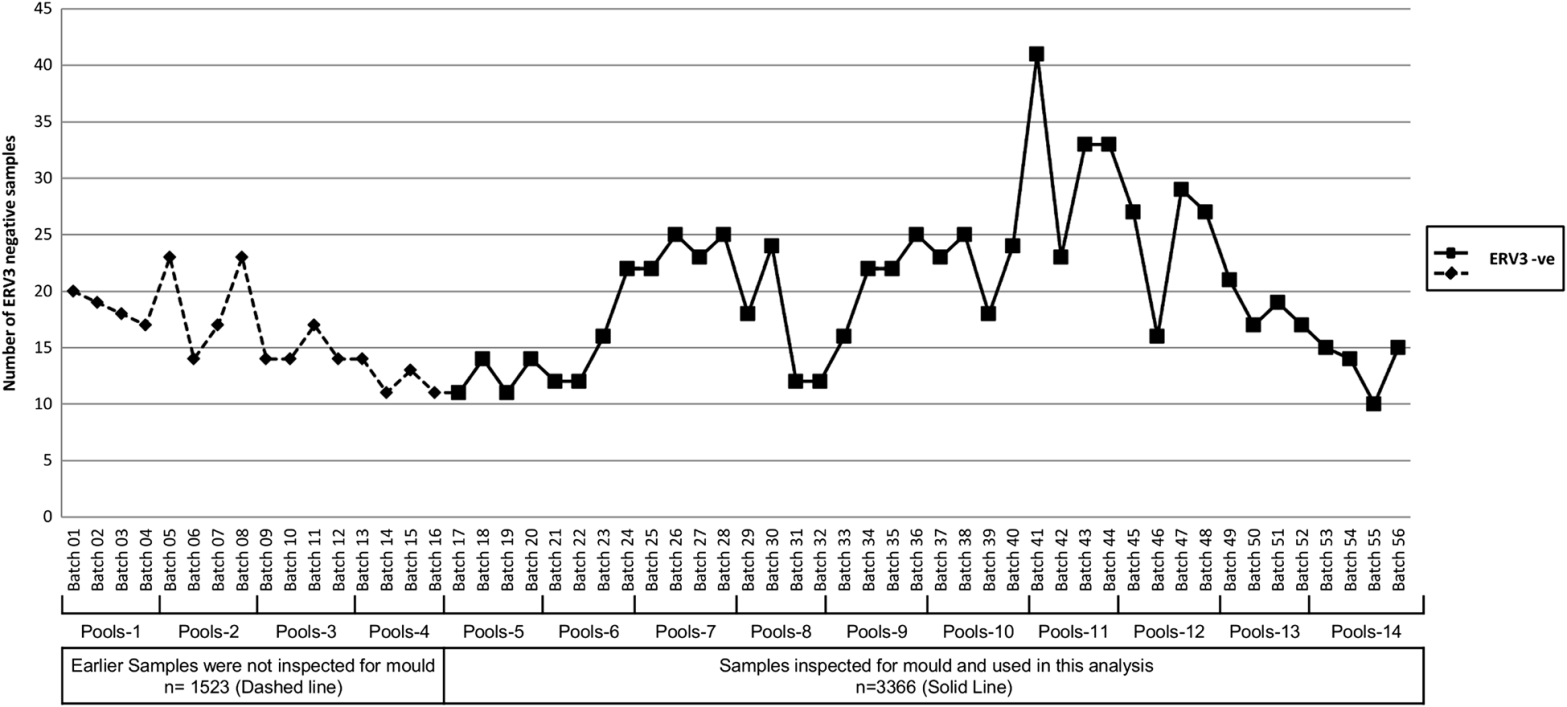
**Number of samples negative for ERV3 during screening of each sample extraction batch (92 samples per batch).** A total of 56 extraction batches were performed in the first 20-months of the study. Each batch was tested for quality control (EHV1/ERV3 PCR), following which every four batches were pooled and pools were screened for respiratory viruses other than rhinovirus. The batches used in the analysis are indicated by the solid line.

For this study, samples that failed EHV1 criteria or were not inspected for mould growth were excluded from the analysis (Figure 1).

### Data analysis

The association between variables of interest and binary outcomes was investigated using mixed effects logistic regression models, with participants included as a random intercept to account for the possibly correlated outcomes within each infant. The association with continuous outcomes was investigated using mixed effects linear regression. When examining the association of mould level with sample quality and respiratory virus detection we conducted both univariate and multivariate analyses, with multivariate analyses adjusting for the potential confounders of the child’s age, gender, relationship of collector to participant (e.g. father, mother or others), season specimen collected, and time from specimen collection to being frozen in the laboratory. Analyses were conducted using Stata statistical software v.11.0 (StataCorp, College Station, TX, USA).

## Results

### Swab samples

Between September 2010 and July 2012, 152 infants were recruited into the study. All participants lived within the greater Brisbane metropolitan area and none were from rural communities. One-hundred and twenty-five recruits remained active study participants up until the date of this analysis. Of the 27 withdrawals, four had moved out of the study area, two others were later deemed ineligible, ten withdrew for personal reasons and eleven were ineligible because they could not fulfill sampling requirements. For the active families, swab return rates were >90% for almost 35,000 child-days of observation. In total, 4933 weekly nasal swab specimens (~510 nasal swabs/month) were batched in 56 (96 well) racks, extracted and tested. The median time from collection to swab arrival in the laboratory was 2 (interquartile range 2–4) days; however 10.9% of swabs were received more than 7-days after their collection.

### Excluded samples

For EHV1 extraction and inhibition testing, 42 (0.81%) DNA extracts failed the EHV1 criteria. The initial 1525 samples were not inspected for mould growth during the early stages of the study and therefore were excluded from further analysis.

### ERV3 detection

Of the remaining 3366 samples, there were 2718 (80.7%) samples positive for ERV3 with PCR amplification Ct values ranging from 23–45 (median 36) cycles. Overall, ERV3 was not detected in 649 (19.2%) samples. During the first 8-months of batching and screening conducted in the laboratory, the number of ERV3 negative samples ranged from 11 to 25 in each 92 extraction run with a median of 17 negative samples per run (Figure 1). However, following a cluster of samples negative for ERV3 (Figure 1; batches 41, 43, 44) we contacted parents and reminded them of the optimal swab collection technique they had been shown at enrolment of their baby. After this feedback the numbers of ERV3 negative samples declined.

### Respiratory viruses detected

At least one respiratory virus was detected in 885 (26.2%) samples. Dual or multiple virus detections were observed in 105 (2.14%) samples. RV was the most common virus detected, being present in almost 20% of specimens, followed by human bocavirus, human polyomavirus KIPyV, respiratory syncytial viruses and human adenoviruses (Table 2).

**Table 2:** Results for respiratory viruses screening from 3366 parent collected nasal swab specimens between July 2011 and July 2012 and fulfilling the EHV1 criteria

**Virus**	**No. of infants**	**No. of samples**	**% of all samples**
**RV**	105	726	21.57
**HBoV**	26	46	1.37
**KIPyV**	17	41	1.22
**HAdV**	23	30	0.89
**RSV-A**	26	30	0.89
**WUPyV**	13	28	0.83
**HCoV NL63**	12	16	0.48
**IV-B**	11	11	0.33
**HCoV 229E**	3	6	0.18
**PIV 1**	6	6	0.18
**HMPV**	5	5	0.15
**PIV3**	3	3	0.09
**HCoV HKU1**	3	3	0.09
**IV-A**	2	2	0.06
**RSV B**	2	2	0.06
**HCoV OC43**	1	1	0.03
**PIV 2**	0	0	0

HAdV, Human adenovirus; HBoV, Human bocavirus; HCoV, Human coronavirus; HMPV, Human metapneumovirus; IV, Influenza virus; PIV, Parainfluenza virus; PyV, Polyomavirus; RSV, Respiratory syncytial virus, RV, Rhinovirus.

### Mould

Of 3366 swab samples visually inspected for mould, 99 (2.9%) had high, 252 (7.5%) medium and 411 (12.2%) had low levels present, while 2604 swabs (77.4%) had no visible signs of mould. The mean (standard deviation) time from collection until being frozen in the laboratory for samples with no observed mould was 2.9 (3.0) days. In comparison for low level mould it was 4.9 (3.6) days (crude mean difference compared with no mould; 95% confidence interval (CI) = 1.7; 1.4 – 2.1 days), for medium level mould it was 7.4 (4.9) days (3.9; 3.4 – 4.3), and for high level mould 11.4 (10.7) days (7.1; 6.4 – 7.8). The mean difference in time from collection until being frozen between each mould group and the no mould group was statistically significant (P < 0.001 for each comparison). A significant association was also observed between mould and season. In specimens collected in summer, mould was observed on 28.2% of swabs. In comparison mould detection rates were 31.0% in spring (crude odds ratio (OR); 95% CI = 1.08; 0.87 – 1.34), 15.8% in autumn (0.47; 0.37 – 0.59) and 13.7% in winter (0.40; 0.29 – 0.53). When considering samples that contained mould, there was no statistically significant association between season and level of mould.

Fungal identification was achieved for 48 of 70 swabs subjected to PCR and DNA sequencing (Table 3). A diverse range of species was observed with *Epicoccum nigrum* and *Cladosporium cladosporioides* the most prevalent.

**Table 3:** Species detected in 70 samples with different levels of fungal growth

**Species**	**Number detected**	**(high; medium; low; no visible mould)**
*Epicoccum nigrum*	15	(7,2,4,2)
*Cladosporium cladosporioides*	7	(3,3,1,0)
*Aureobasidium pullulan*	4	(1,1,2,0)
*Cryptococcus flavescens*	3	(1,2,0,0)
*Alternaria alternata*	2	(1,1,0,0)
*Alternaria tenuissima*	1	(0,0,1,0)
*Aspergillus westerdijkiae*	1	(0,0,1,0)
*Candida parapsilosis*	1	(0,1,0,0)
*Cladosporium silenes*	1	(0,0,0,1)
*Cladosporium tenuissimum*	1	(0,1,0,0)
*Cladosporium uredinicola*	1	(1,0,0,0)
*Cochliobolus lunatus*	1	(0,1,0,0)
*Curvularia brachyspora*	1	(0,1,0,0)
*Curvularia trifolii*	1	(0,1,0,0)
*Leptosphaerulina australis*	1	(0,1,0,0)
*Paraphaeosphaeria sp*	1	(1,0,0,0)
*Penicillium fellutanum*	1	(0,0,1,0)
*Penicillium oxalicum*	1	(0,1,0,0)
*Penicillium polonicum*	1	(0,0,0,1)
*Penicillium spinulosum*	1	(0,0,1,0)
*Phoma herbarum*	1	(0,0,1,0)
*Rhodotorula slooffiae*	1	(0,1,0,0)
**Total**	**48**	

### ERV3, visible mould and respiratory virus detection

**Figure 2:**
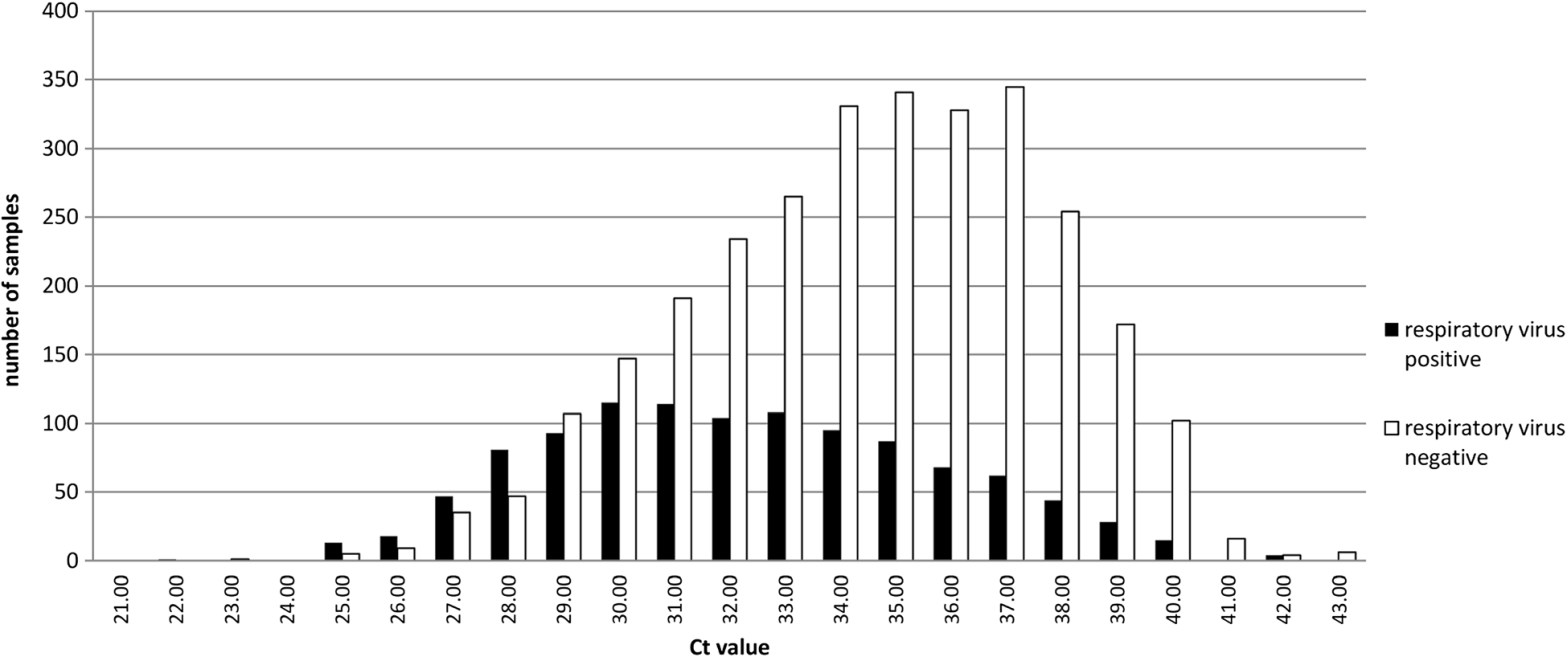
**Comparison between average ERV3 cycle threshold (Ct) values in respiratory virus positive (dark bars) versus negative (light bars) samples.** In ERV3-positive samples, the average ERV3-Ct values (32.8) in samples positive for any virus was significantly lower than the average ERV3-Ct values (35.4) in samples negative for all viruses (difference = 2.6, 95% confidence interval 2.3-2.9).

Of the 2718 samples that were ERV3 positive, 810 (37.2%) had at least one respiratory virus detected by PCR. In contrast, the respiratory virus detection rate in ERV3 negative samples was significantly lower (75/649, 11.5%; crude odds ratio (OR) = 0.35; 95% CI 0.27-0.44) when ERV3 was absent in swab specimens. We also observed that among ERV3 positive swabs, the average ERV3 Ct value for samples positive for any respiratory virus (32.8 cycles) was significantly lower (indicating greater ERV3 load) than the average Ct value (35.4) in samples negative for all viruses (crude difference = 2.0, 95% CI 1.4 – 2.6; Figure 2). Moreover, there was a significant difference in ERV3 Ct values (P = 0.001) in samples that had single respiratory virus detection (average = 33.01) comparing with samples that had multiple respiratory viruses detection (average = 31.27).

Of the 762 samples with visible mould, 529 (69.4%) were positive for ERV3, which was significantly lower than rates in samples without visible mould (84.0%; crude OR = 0.35, 95% CI 0.28-0.43). The proportion of samples with visible mould and positive respiratory virus testing (178/762; 23.4%) was significantly lower than that of samples without mould (707/2606; 27.1%; crude OR = 0.70, 95% CI 0.57-0.86).

Table 4 examines the association between ERV3 and respiratory virus detection and potential explanatory and confounding variables. ERV3 positive sample rates increased with age, varied by season and declined with increasing mould levels and time taken for samples to reach the laboratory and to be frozen. Similarly, respiratory virus detection rates increased with age, specimen collection outside the summer months, and time taken to reach the laboratory, while decreasing as visible mould levels in samples reaching the laboratory increased.

**Table 4:** ERV3 and respiratory virus positive samples detected by polymerase chain reaction assays in 3366 parent collected nasal swab specimens

**Variable**		**No. samples (%)**	**ERV3 Positive**			**Respiratory virus positive**		
			**No. samples (%)**	**Univariate**	*Multivariate	**No. samples (%)**	**Univariate**	*Multivariate
				**OR (95% CI); P value**	**OR (95%); P value**		**OR (95% CI); P value**	**OR (95%); P value**
**Age (months)**	***< 6***	1293 (38.4%)	995 (77.0)	1	1	208 (16.1)	1	1
	***6- <12***	1295 (38.5%)	1061 (81.9)	1.20 (0.94-1.53); 0.15	1.28 (0.98-1.68); 0.07	411 (31.7)	2.59 (2.07-3.24); <0.001	2.38 (1.89-3.01); <0.001
	***≥12***	778 (23.1%)	662 (85.1)	1.49 (1.06-2.10); 0.02	1.93 (1.27-2.93); 0.002	266 (34.2)	2.98 (2.26-3.92); <0.001	2.16 (1.57-2.99); <0.001
**Gender**	***Male***	1647 (48.9%)	1335 (81.1)	1	1	461 (28.1)	1	1
	***Female***	1719 (51.06%)	1383 (80.4)	0.81 (0.54-1.22); 0.32	0.87 (0.58-1.29); 0.48	424 (24.7)	0.82 (0.60-1.12); 0.21	0.83 (0.61-1.12); 0.23
**Collector**	***Mother***	2845 (84.5%)	2307 (81.1)	1	1	766 (26.9)	1	1
	***Father***	441 (13.1%)	342 (77.6)	0.91 (0.66-1.27); 0.60	0.87 (0.62-1.22); 0.42	109 (24.7)	0.94 (0.70-1.26); 0.67	0.88 (0.65-1.19); 0.41
	***Research staff***	45 (1.3%)	40 (88.9)	2.71 (1.00-7.36); 0.05	1.76 (0.65-4.81); 0.27	3 (6.7)	0.24 (0.07-0.79); 0.02	0.36 (0.11-1.21); 0.10
	***Other***	35 (1.0%)	29 (82.9)	1.31 (0.49-3.51); 0.59	1.39 (0.46-4.16); 0.56	7 (20.0)	0.72 (0.30-1.74); 0.47	0.87 (0.35-2.13); 0.76
**Season**	***Summer***	926 (27.5%)	729 (78.7)	1	1	178 (19.2)	1	1
	***Autumn***	1059 (31.5%)	802 (75.7)	0.90 (0.71-1.13); 0.37	0.74 (0.58-0.96); 0.02	304 (28.7)	1.99 (1.59-2.49); <0.001	1.74 (1.38-2.20); <0.001
	***Winter***	541 (16.1%)	482 (89.1)	2.63 (1.87-3.70); <0.001	2.41 (1.67-3.49); <0.001	198 (36.6)	3.06 (2.36-3.97); <0.001	2.63 (2.01-3.45); <0.001
	***Spring***	840 (25.0%)	705 (83.9)	1.39 (1.07-1.79); 0.01	1.50 (1.13-1.99); 0.005	205 (24.4)	1.27 (1.00-1.61); 0.05	1.43 (1.11-1.84); 0.005
**Mould**	***None***	2604 (77.4%)	2189 (84.1)	1	1	707 (27.2)	1	1
	***Low***	411 (12.2%)	308 (74.9)	0.47 (0.36-0.62); <0.001	0.69 (0.52-0.93); 0.01	97 (23.6)	0.73 (0.56-0.95); 0.02	0.81 (0.61-1.07); 0.14
	***Medium***	252 (7.5%)	163 (64.7)	0.27 (0.20-0.37); <0.001	0.47 (0.33-0.66); <0.001	60 (23.8)	0.70 (0.50-0.96); 0.03	0.70 (0.49-0.99); 0.05
	***High***	99 (2.9%)	58 (58.6)	0.20 (0.13-0.33); <0.001	0.40 (0.24-0.66); <0.001	21 (21.2)	0.57 (0.34-0.96); 0.04	0.53 (0.31-0.93); 0.03
**Time to reach Laboratory (days)**	***0*-*3***	2281 (67.8%)	1983 (86.9)	1	1	587 (25.7)	1	1
	***4*-*7***	723 (21.5%)	513 (71.0)	0.32 (0.25-0.40); <0.001	0.39 (0.30-0.50); <0.001	187 (25.9)	0.96 (0.78-1.18); 0.69	1.03 (0.82-1.29);0.80
	**>** ***7***	362 (10.8%)	222 (61.3)	0.17 (0.13-0.24); <0.001	0.24 (0.17-0.34); <0.001	111 (30.7)	1.16 (0.89-1.52); 0.28	1.42 (1.05-1.94); 0.02

*Adjusted for all variables in the Table.

## Discussion

The ORChID project is an ongoing comprehensive community-based study using PCR assays to detect respiratory viruses in anterior nasal swab specimens taken weekly by parents from their infants throughout the first 2-years of life. This requires parents following a standardized protocol of obtaining swabs regularly and mailing them promptly to our laboratory. However, we have observed that suboptimal sample collection as determined by ERV3 detection and presence of visible mould in swab samples reaching the laboratory can negatively affect sample quality and potentially respiratory virus detection.

The data from the first 20-months of our longitudinal study indicate that respiratory virus detection is associated with the ERV3 load in nasal swab specimens. Swabs negative for ERV3, presumably from sub-optimal collection, had reduced respiratory virus detection rates compared with samples containing ERV3. Furthermore, in those specimens positive for ERV3, a higher ERV3 load was associated with a higher likelihood of respiratory virus detection. Overall, this shows the importance of measuring human DNA as a marker for epithelial cells in swab samples, which if tested and monitored in real time during the study, can identify problems associated with collection that can be addressed quickly. This is illustrated in the current study when a sudden increase in ERV3 negative samples was observed. Parents were contacted and reminded about sample collection protocols following which there was a decline in ERV3 negative sample rates towards baseline levels.

We were also concerned at finding mould on some samples, which occurred despite the commercial swab tubes containing antifungal agents. Most fungal species identified in the swabs were saprophytic, and the most common fungus found, *Epicoccum nigrum*, is a known contaminant of clinical specimens [36]. The relationship between fungal airspora counts and meteorological conditions is complex and impacts at the species level [37]. In Brisbane, *Cladosporium and Alternaria* airspora are detected commonly throughout the year, but as with *Epicoccum*,sp their levels peak during the warmer, humid months. Other factors, such as rainfall and wind speed, can also influence fungal airspora composition [37,38]. In our study, mould was associated mainly with longer time intervals between taking swabs and their arrival at the laboratory. However, this was especially evident during the warm, humid spring and summer months, which leads us to speculate that fungal contamination occurred during sample collection and was influenced by the aforementioned environmental factors. Unfortunately, we could not explore this further as it was beyond the scope of the present study. In addition, while mould growth proved to be an issue in the subtropical climate of Brisbane, this may be less of a problem in more temperate climates with lower temperatures and humidity levels.

We now remind parents regularly to mail swabs promptly after collection. Of interest however, was that respiratory virus detection rates were not affected by prolonged transport times, but in fact appeared to increase with time taken to reach the laboratory. While the observed increase was unexpected and may have occurred simply by chance, it is plausible that viral nucleic acids were protected to some extent by being encapsulated within the viral capsid, and by using viral transport medium in the swabs.

Fungi were found to be associated with both reduced ERV3 detection and, at high levels, reduced significantly respiratory virus detection. At least three points emerge from this study. First, although swabs may contain antimicrobial agents, the risk of fungal and potentially bacterial contamination may still arise. Second, the times between swab collection and laboratory arrival should be monitored and feedback provided if delays occur. Finally, if delays are expected swabs should be placed in the household refrigerator until mailed to the laboratory [20].

## Conclusion

We found that ERV3 as a marker for human DNA and epithelial cells was also an important indicator of sample quality for our study. For community-based investigations similar to our own, real-time sample processing and ERV3 detection can facilitate rapid interventions to maintain sample quality and to optimize respiratory virus detection. Indeed, this may have broader implications since nasal swabs are beginning to replace the traditional, but more invasive nasopharyngeal swab or aspirate sampling techniques in hospitals and clinics, especially following the 2009 influenza pandemic [17]. Thus, similar ERV3 testing strategies could be used by diagnostic laboratories to improve or monitor sample collection quality for optimal respiratory virus detection. Finally, the potential problem of visible mould contamination of swabs taken during community-based studies can be minimized by ensuring samples are transported promptly to the laboratory.

## Abbreviations

ARI: Acute respiratory infection; Ct: Cycle threshold; DNA: Deoxyribonucleic acid; EHV1: Equine herpes virus 1; ERV3: Endogenous retrovirus 3; ITS: Internal transcribed spacer; OR: Odds ratio; ORChID: Observational research in childhood infectious diseases; PCR: Polymerase chain reaction; PyV: Polyomaviruses; RV: Rhinovirus.

## Competing interests

The authors declare that they have no competing interest.

## Authors’ contributions

Conceived and designed the experiments: AA, DW, SB, SL, TS, KG. Performed the experiments: AA, DW, SB. Analyzed the data: RW, AA, SL. Contributed reagents/materials/analysis tools: AA, DW, SB, SL, TS, KG. Wrote the manuscript: AA, DW, SL, KG. Approved the final manuscript: AA, DW, SB, SL, RW, MN, TS, KG. All authors read and approved the final manuscript.

## Pre-publication history

The pre-publication history for this paper can be accessed here:

http://www.biomedcentral.com/1471-2334/14/15/prepub
